# Metasurface‐Assisted Wireless Communication with Physical Level Information Encryption

**DOI:** 10.1002/advs.202204558

**Published:** 2022-10-17

**Authors:** Yilin Zheng, Ke Chen, Zhiyuan Xu, Na Zhang, Jian Wang, Junming Zhao, Yijun Feng

**Affiliations:** ^1^ School of Electronic Science and Engineering Nanjing University Nanjing 210023 P. R. China

**Keywords:** dual‐band, information encryption, programmable metasurface, wireless communication

## Abstract

Since the discovery of wireless telegraphy in 1897, wireless communication via electromagnetic (EM) signals has become a standard solution to address increasing demand for information transfer in modern society. With the rapid growth of EM wave manipulation technique, programmable metasurface (PM) has emerged as a new type of wireless transmitter by directly modulating digital information without complex microwave components, thus providing an alternative to simplify the conventional wireless communication system. However, the challenges of improving information security and spectrum utilization still exist. Here, a dual‐band metasurface‐assisted wireless communication scheme is introduced to provide additional physical channels for the enhancement of information security. The information is divided into several parts and transmitted through different physical channels to accomplish information encryption, greatly reducing the possibility of eavesdropping. As the proof of concept, a dual‐channel and high‐security wireless communication system based on a 1‐bit PM is established to simultaneously transmit two different parts of a picture to two receivers. Experiments show that the transmitted picture can be successfully retrieved only if the received signals of different receivers are synthetized as predefined. The proposed scheme provides a new route of employing PM in information encryption and spectrum utilization of wireless communication.

## Graphical abstract

A metasurface‐assisted wireless communication prototype system with additional physical channels to enhance the information security is proposed here. The information is divided into several parts and transmitted simultaneously through different frequency‐channels to avoid eavesdropping. The system demo proves that a colour picture can be successfully encrypted and retrieved with high reliability.

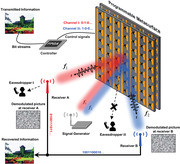

## 1 Introduction

Metasurfaces, 2D artificial structures composed of judiciously engineered subwavelength inclusions, have showcased exotic abilities to arbitrarily manipulate the amplitude, phase and polarization of electromagnetic (EM) waves in both transmission and reflection geometries.^[^ ^1^, ^2^, ^3^, ^4^ ^]^ Many intriguing physical phenomena and devices have emerged based on metasurface techniques, such as invisibility cloaks,^[^ ^5^, ^6^ ^]^ wave plates,^[^ ^7^ ^]^ imaging systems,^[^ ^8^, ^9^ ^]^ and other functional devices.^[^ ^10^, ^11^, ^12^, ^13^ ^]^ Moreover, by introducing active materials or components such as graphene, phase‐change material, and electrically driven diode/varactor into metasurfaces,^[^ ^14^, ^15^, ^16^, ^17^, ^18^ ^]^ it becomes possible to dynamically realize versatile EM responses via external control, thus eliciting an inevitable transition from static to dynamic wavefront tailoring for advancing the field forward. For example, the programmable metasurfaces (PMs) offer an innovative method to combine metasurface with the digital systems.^[^ ^19^ ^]^ In such a way, the output wave functionalities can be switched in real‐time by simply changing the coding sequences through a computer‐controlled hardware controller, thus implementing many intriguing devices and applications such as dynamic imager,^[^ ^20^ ^]^ tunable beam shaper,^[^ ^21^ ^]^ and active metalens.^[^ ^22^ ^]^ Especially, the programmable metasurfaces can realize direct signal processing by controlling the EM responses (such as amplitude, phase, etc.) in real time, making it a new type of transmitter for wireless communication.^[^ ^23^, ^24^, ^25^, ^26^, ^27^, ^28^, ^29^, ^30^ ^]^

Compared with traditional wireless communication transmitters, the metasurface‐assisted transmitter can directly modulate the phase, amplitude, or frequency of baseband signals without mixers and other microwave components, largely simplifying the wireless communication system. To date, several modulation schemes have been applied to metasurface‐assisted wireless communication transmitters, including binary frequency‐shift keying (BFSK),^[^ ^23^ ^]^ quadrature phase‐shift keying (QPSK),^[^ ^24^ ^]^ and quadrature amplitude modulation (QAM).^[^ ^27^ ^]^ However, most of them only have single operation frequency band, lacking adaptability and scalability that may restrict their practical applications. Actually, a single operation frequency may not meet the demand of larger information capacity or make full use of spectrum resources, whereas both of which are needed in the fast development of fifth generation (5G) wireless communication and beyond. Only very recently, attempts on frequency‐division multiplexing was proposed to transmit information at two harmonic frequencies,^[^ ^28^ ^]^ but in fact, the frequencies are too close (about several megahertz) to prevent interference with each other, and they could be easily shielded or eavesdropped at the same time. Thus, achieving desired multichannels in different frequencies is still challenging and also in great need for the development of metasurface‐assisted new‐architecture wireless communication.

On the other hand, physical layer security using the uniqueness and reciprocity of the physical channel to implement information encryption such as wiretap coding, relay cooperation interference, beamforming, and physical layer key generation, has emerged as a promising method to enhance information security in wireless communication.^[^ ^31^, ^32^, ^33^, ^34^, ^35^ ^]^ In metasurface‐assisted wireless communication system, physical channels represent metasurface’ physical functions that can be modulated to transmit information, for example, spatial beamforming. The basic wiretap channel model consists of one transmitter, one legitimate receiver, and one eavesdropper.^[^ ^36^ ^]^ To ensure information security, legitimate receiver channels should have the maximum signal‐to‐noise ratio (SNR) while eavesdropper channels should be suppressed with the minimum SNR.^[^ ^37^, ^38^ ^]^ Therefore, for single‐frequency metasurface transmitters, the legitimate receiver should be in the direction of the beam generated by the metasurface while the eavesdropper out of beam range. However, a special case frequently appears that the eavesdropper and the legitimate receiver are in the same direction in many practical scenarios, which leads to high correlation of their channel responses and the transmitted information may easily be intercepted. On the contrary, if there are additional physical channels (e.g., frequency), secret sharing can be adopted to solve the cryptographic task by splitting a secret among multiple shareholders,^[^ ^39^, ^40^ ^]^ so that the eavesdropping of a single channel will not leak any information about the shared secret.

To solve the above problems, here we explore dual‐band programmable metasurface to provide additional physical channels for the tremendous enhancement of information security. The information is divided into different parts and transmitted in different physical channels for the information encryption. In this case, even if the eavesdropper and the receiver are at the same position, the correct information cannot be cracked from a single physical channel, thus largely improving the information security. Moreover, as the linking channels are determined by the phase profile on the metasurface, dynamic wireless communications can be achieved, such as target tracking, multitarget communication, etc. Considering the practical applications, the two operation frequency bands of the tunable metasurface are centered at 2.4 GHz (*f*_1_) and 5 GHz (*f*_2_), which are the working frequencies of the commercial dual‐band WiFi network. As a proof of concept, a high‐security binary amplitude‐shift keying (BASK) metasurface‐based wireless communication system is constructed and experimentally verified to transmit the target picture to legitimate receiver. The picture information is divided into two parts and directly encoded onto the incident waves by dynamically switching the working state of the metasurface. At the same time, the received binary symbols at the two receivers are synthetized in real time to obtain the transmitted information, which cannot be retrieved by either of the receivers. The measured bit error rate (BER) is as low as 8.1 × 10^−4^, and the transmission rate can reach up to 4.167 Mbps for this prototype. The proposed scheme provides a new perspective on the role of programmable metasurface in information encryption and spectrum utilization of wireless communication, which can be transformed to other frequencies for a wide range of applications.

## 2 Concept and the Operation Principle

**Figure 1:**
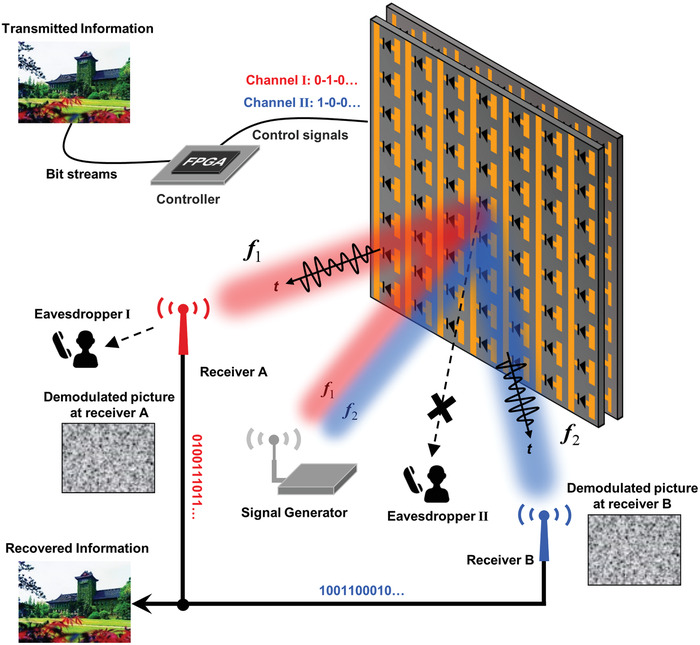
The conceptual illustration of the high‐security metasurface‐assisted wireless communication system. By dividing the information of the transmitted picture into two parts, different control‐voltage sequences are applied from the FPGA to the PIN diodes on the metasurface to transmit different information via physical channels of different frequencies. The demodulated information of receiver A and receiver B should be synthesized in order to obtain the correct transmitted picture while the recovered picture at individual receiver is meaningless, thus protecting the information security.

The schematic of the high‐security metasurface‐assisted wireless communication is depicted in **Figure** **1**. It contains four major parts: carrier wave, PM‐based modulator, field programmable gate array (FPGA) hardware control platform, and demodulator. The information (e.g., text, picture, or video) is transformed into binary bit‐stream and divided into several parts according to the physical channel number. For simplicity, we consider a single beam generation in each of the two operation frequencies (*f*_1_ and *f*_2_), so the bit‐stream is divided into two parts. Then, the two bit‐streams are synchronously encoded onto the reflections of the incident waves by a PM through switching the output voltage signals of the FPGA controller. Hence, each of the modulated signals only contains parts of the shared secret, which will be interpreted as unreadable codes at each receiving terminal. Finally, legitimate receiver can receive the transmitted information by combining all of the received information.

In the metasurface‐assisted direct wireless communication, the information encoding and signal modulation are directly realized by the metasurface on the modulator's side. When the metasurface is normally illuminated by the monochromatic wave *E*_*i*_(*t*) at *f* = *f*_c_, the output response — the reflection wave can be represented as

(1) $$E_{r}\left(t\right)=\Gamma \left(t\right)E_{i}\left(t\right)=A\left(t\right)e^{j\left[2\pi f\left(t\right)\times t+\varphi \left(t\right)\right]}e^{j2\pi f_{c}t}$$

in which *A*(*t*), *f*(*t*), and *φ*(*t*) are the amplitude, frequency, and phase of the reflection coefficient Γ(*t*). From Equation (1), it is easy to obtain amplitude, frequency, and phase modulation signals that resides on the carrier wave. In digital modulation, the analog waveforms should be discretized to match the digital information. Thus, the reflection coefficient Г(*t*) of the metasurface could be simplified as^[^ ^41^ ^]^

(2) $$\Gamma \left(t\right)=\Gamma_{m}g\left(t\right),0\leq t\leq T,\Gamma_{m}\in M$$

where {Г_*m*_} is the binary data sequence of Г(*t*), and it can be either real or complex value according to the modulation type. *g*(*t*) is the basic pulse shaping function, and *T* is the message symbol duration. *M* is a set of constellation points with cardinal number *card*(*M*). Each message symbol Г_*m*_ can be mapped to log_2_ *card*(*M*) bits digital information.

In general, the modulation schemes of the metasurface‐assisted wireless communication system mainly contain three basic kinds, namely the frequency, phase and amplitude modulations. Each one has its unique features, for example, amplitude modulation has the advantages of smaller distortion and simpler implementation. Here, we consider amplitude modulation for demonstration of the high‐security metasurface‐assisted direct wireless communication; however, other modulation methods can also be realized by the proposed metasurface. For amplitude modulation, each message symbol can be expressed as

(3) $$\Gamma_{m}=A_{m}e^{j\varphi }$$

where *A*_*m*_ is the different reflection amplitude of the metasurface, corresponding to the amplitude of the constellation point. *φ* is the reflection phase that is identical for different discrete states. Thus, with a certain cardinal number *card*(*M*), the constellation point set *M* that Г_*m*_ belongs to can be defined as

(4) $$\begin{array}{ccc} \Gamma_{m}\in M & = & \left\{A_{0}e^{j\varphi },A_{1}e^{j\varphi },\cdots ,\;A_{\mathrm{card}\left(M\right)-1}e^{j\varphi }\right\},\\ & & m=1,2,\cdots ,\mathrm{card}\left(M\right)-1 \end{array}$$

Each element of *M* can be mapped to a digital code in a log_2_ *card*(*M*)‐bit amplitude modulation scheme. Taking BASK scheme as an example, *card*(*M*) equals 2 and a mapping relationship between Г_*m*_ and digital codes in a BASK system can be established as

(5) $$\Gamma_{0}=A_{0}e^{j\varphi }\Leftrightarrow '0',\Gamma_{1}=A_{1}e^{j\varphi }\Leftrightarrow '1'$$

This relationship indicates that the tunable reflection coefficient Г_*m*_ of the programmable metasurface can be encoded by log_2_ *card*(*M*)‐bit binary digits. Hence, one of the key steps to realize a wireless communication system via amplitude modulation is to design a metasurface with a dynamic amplitude response but constant phase response.

To that end, a dual‐band PM with independent reconfigurable far‐field pattern at each frequency (*f*_1_ and *f*_2_) should be designed, of which the constituent meta‐atom should have opposite reflection phase to provide dynamic beam generation. By applying different bias voltages to the PIN diodes embedded in each meta‐atom, the reflection beam of the PM can be controlled in real time based on elaborately designed coding matrices, thus transmitting binary symbols based on amplitude modulation. The coding matrices of the metasurface are designed to generate beams with different pointing directions and beam number, thus producing amplitude variation at a certain radiation angle to enact the amplitude modulations and construct different communication channels at various directions. In other words, the metasurface is capable of reconfiguring the wireless propagation environment according to the location of the receiver. Moreover, the signal modulation is directly achieved by the metasurface, which has simpler architecture compared with conventional wireless communication with complex RF components.

At the demodulator's side, receivers are located in the coverage area of the beams to receive the modulated baseband signals in two physical channels, for example, receiver A for channel working with *f*_1_ and receiver B for channel working with *f*_2_. The original information will be retrieved only if the received information at receiver A and receiver B are synthetized as predefined. Each receiver recovers only part of the original information and presents a meaningless message. In addition, it is worth noting that the locations of receiver A and B can be arbitrarily designed via controlling the metasurface, and even they are in the same direction, they still communicate with different physical channels via *f*_1_ and *f*_2_ without interfering with each other.

## 3 Design of Programmable Metasurface and the Coding Matrix

**Figure 2:**
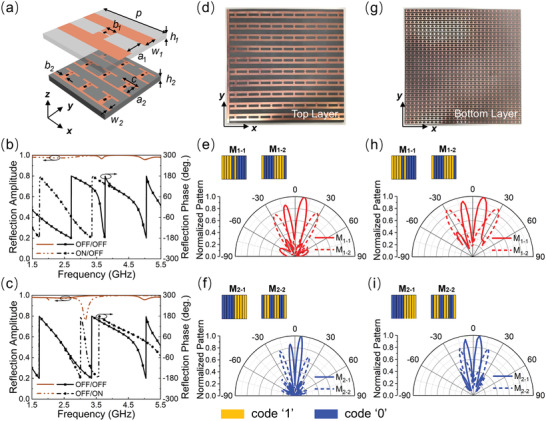
a) The meta‐atom of the programmable metasurface. b) The simulated reflection responses to the “ON” and “OFF” states of the PIN diodes on the top layer when the PIN diodes on the bottom layer are in the “OFF” state. c) The simulated reflection responses to the “ON” and “OFF” states of the PIN diodes on the bottom layer when the others are in the “OFF” state. d) The top layer of the fabricated PM. The simulated far‐field patterns of the optimized coding matrices e) *M*_1–1_, *M*_1–2_ at 2.4 GHz and f) *M*_2–1_ and *M*_2–2_ at 5 GHz in *yoz* plane. g) The bottom layer of the fabricated PM. The measured far‐field patterns of the optimized coding matrices h) *M*_1–1_, *M*_1–2_ at 2.15 GHz and i) *M*_2–1_ and *M*_2–2_ at 5.34 GHz in *yoz* plane. Receiver A (at 2.4 GHz) is located at *θ* = −12° and receiver B (at 5 GHz) is located at *θ* = 5°.

To construct the proposed metasurface‐assisted wireless communication system, a double‐layered meta‐atom loaded with PIN diodes is employed to achieve dual‐band tunable phase responses as mentioned above. In **Figure** **2**a, an I‐shaped metallic pattern with a gap cut in the middle wire and connected by a PIN diode is selected as the top layer, which is printed on an ungrounded dielectric layer with thickness of *h*_1_ = 3 mm. The bottom layer is made of a 3×3 array of identical patterns printed on the dielectric substrate with a thickness of *h*_2_ = 4 mm, which is separated from the top layer by air (thickness of 25 mm). The dielectric layers are made of F4B with a relative permittivity of 2.2 and loss tangent of 0.001. The two metal arms of the I‐shaped patterns (both top layer and bottom layer) are the positive and negative poles, respectively, to provide a direct current (DC) bias voltage for switching the diodes between “ON” and “OFF” states. More details about the biasing network are shown in Figure S1 in the Supporting Information. The meta‐atom has a period of *p* = 48 mm, and other geometric parameters are optimized as *a*_1_ = 10.4 mm, *b*_1_ = 8 mm, *w*_1_ = 9.3 mm, *a*_2_ = 7.2 mm, *b*_2_ = 3.5 mm, *c* = 12.3 mm, and *w*_2_ = 1.3 mm.

To investigate the EM response of the designed meta‐atom, full‐wave simulations are performed by commercial software. The meta‐atom is illuminated by a *y*‐polarized EM wave, with periodic boundary conditions along the lateral sides and Floquet ports along the longitudinal sides. The PIN diode is equivalent to a resistor‐inductor‐capacitor (RLC) series circuit with parameters of *R* = 0.5 Ω and *L* = 0.7 nH for the “ON” state and *L* = 0.5 nH and *C* = 0.24 pF for the “OFF” state. The reflection amplitude and phase response of the meta‐atom are illustrated in Figure 2b,c. It can be observed that a phase difference of 180° at 2.4 GHz can be obtained when the PIN diodes on the top layer are switched between “ON” to “OFF” states while the reflection amplitudes are over 0.95. Similar results can be observed at 5 GHz when the PIN diodes on the bottom layer are switched between “ON” to “OFF” states, as shown in Figure 2c. Moreover, the amplitude and phase responses of the meta‐atom when the PIN diodes on one layer are switched between “ON” to “OFF” states while the PIN diodes on the other layer are kept at “ON” state are also depicted in Figure S2 in the Supporting Information. It's clear that the amplitude and phase responses of each layer of the meta‐atom are kept the same, regardless that the PIN diodes on the other layer are at “ON” or “OFF” state, thus proving the independence of two frequency‐channels.

The dual‐band switchable meta‐atoms are then used as the building blocks to construct the PM, and the fabricated sample are shown in Figure 2d–g. Two layers of the PM are both composed of 10×10 meta‐atoms; thus, each layer has a coding matrix of 10×10 representing the working state of each meta‐atom, with code “1” for “ON” state (bias voltage of 3.3 V) and “0” for “OFF” state (bias voltage of 0 V). Hence, we need to study two coding matrices with a size of 10×10 that can produce two independent far‐field patterns at the two operating frequencies. For simplicity and the consideration of only requiring beam variation in the horizontal plane, the meta‐atoms in each column share the same bias voltage; hence, the coding matrix only have a varying spatial distribution along *y*‐direction. By optimizing the coding matrices of the PM, the main beams at the two operating frequencies can be engineered to point towards the desired directions for the target users. Here, as an illustrative example, two receivers located in different directions are selected in the far‐field region to construct two independent communication channels. We assume that receiver A (at 2.4 GHz) and receiver B (at 5 GHz) are located at directions of azimuth angle *θ* = −12° and *θ* = 5° with respect to the normal of the PM, respectively. Receivers at other directions can also be realized through simply changing the coding matrixes, as depicted in Figure S3 in the Supporting Information. From Equation (5), we can define in the wireless communication that the low power of the beam represents the binary symbol “0” and the high power of the beam represents the binary symbol “1”. Figure 2e presents two simulated far‐field patterns of two optimized coding matrices *M*_1–1_ and *M*_1–2_ at 2.4 GHz, in which the beam of *M*_1–1_ produces a strong peak in the direction of *θ* = −12° that transmits binary symbol “1” to receiver A, while the beam of *M*_1–2_ produces a weak beam in the same direction that transmits binary symbol “0” to receiver A. Similarly, the far‐field patterns of *M*_2–1_ and *M*_2–2_ are also optimized to transmit binary symbols “1” and “0” to receiver B at higher frequency channel, respectively. As shown in Figure 2f, in the direction of *θ* = 5°, the scattering beam of *M*_2–1_ has a strong peak, while the power of beam *M*_2–2_ is very low. In this case, the power intensities of beams at 2.4 and 5 GHz are responsible for transmitting the binary symbols to receivers A and B. Besides, the proposed metasurface is designed to work in a point‐to‐point communication scenario with narrow‐beam range as an illustrative example. It can be extended to the point‐to‐multipoint scenario with wide‐beam range through delicately designing the coding matrixes.

Experiments are carried out to measure the beam scattering properties of the fabricated programmable metasurface. Figure 2h–i displays the measured far‐field patterns when the metasurface is biased with *M*_1–1_ ∼ *M*_2–2_. The measured results at two operation frequencies both have a very slight frequency shift compared with the simulated results, which is mainly due to the fabrication and assembly tolerance, as well as the additional parasitic capacitance and inductance of the electronic elements under the illumination of EM waves. Nevertheless, the far‐field amplitude difference between the two beams are still larger than 0.8 at both 2.15 and 5.34 GHz, which are sufficient to construct the proposed metasurface‐assisted wireless communication system. Besides, the minimum amplitude difference of the two beams to ensure that the high‐level and low‐level receiving signals can be correctly judged as different symbols is about 0.2 on the basis of current experiment conditions. The measured reflection phases and amplitude of the metasurface with uniform bias voltages are shown in Figure S4 (Supporting Information), where 180° phase differences are observed at both frequencies by switching the working states of the PIN diodes loaded on the metasurface. Overall, the good performance of the far‐field patterns guarantees the feasibility of the proposed information‐encoding scheme, which is also the cornerstone of the wireless communication system.

## 4 Experimental Validation of the Wireless Communication System

**Figure 3:**
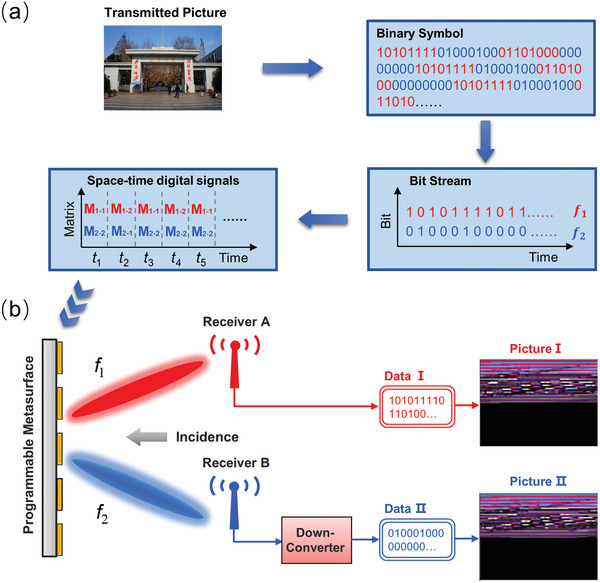
Schematic diagrams of the information decryption processing: a) transmitting terminal, b) receiving terminal, in which the receiving picture I and picture II are measured in the experiment.

**Figure** **3** presents the diagram of the transmitting and receiving process in direct wireless communication with the fabricated 1‐bit PM serving as the transmitter. First, the transmitted information (for example, a picture) is translated into a sequence of binary symbols (for example, “10 101 111…”) via replacing each pixel with 24‐bit binary data sequentially, as shown in the left panel of Figure 3a. Subsequently, the whole binary symbols are divided into many groups and each group, denoted by a group serial number *Q* (*Q* is an integer, *Q* ≥ 1), has *n* (*n* is an integer, *n* ≥ 1) binary symbols. For example, group 1 (*Q* = 1) contains 1^st^ ∼ *n* ^th^ bits, group 2 (*Q* = 2) contains *n*+1^th^ ∼ 2*n* ^th^ bits, and group *Q* contains (*Q*−1)×*n*+1^th^ ∼ *Q*×*n* ^th^ bits. Then, the groups are divided into two parts as the red and blue data to composite bit streams for different physical channels. For example, binary symbols in odd (*Q* = 1, 3, 5…) groups composite the bit stream for *f*_1_ channel and the others composite the bit stream for *f*_2_ channel, as shown in the right panel of Figure 3a. After that, the two bit streams are mapped to the corresponding coding matrices (for example, “*M*_1–1_ − *M*_1–2_ − *M*_1–1_…” and “*M*_2–2_ − *M*_2–1_ − M_2–2_…” as shown in the left panel), which are previously optimized in Figure 2 for routing the desired binary symbols to receivers. Finally, a series of coding matrices are applied to drive the PM in real time through FPGA platform. Hence, the carrier waves of *f*_1_ and *f*_2_ illuminated onto the PM sample are directly modulated by the time‐variant beams, which are then received by the target users. By the way, the group dividing method illustrated in Figure 3 is just an example of verification of the proposed wireless communication system and is not limited to that described here.

Figure 3b describes the receiving process for the two receivers. For the convenience of sampling and demodulation, the received signal of receiver B (*f*_2_) is converted to a lower frequency *f*_3_ through a down converter. Then, the two signals of receiver A and receiver B are transmitted to different universal software radio peripherals (USRPs) to retrieve the carrying messages. A threshold value of power is set up in advance to make threshold decisions of the BASK modulated baseband signals. When the detected power is higher (lower) than the threshold value, the demodulator judges the received digital information as the binary symbol “1” (“0”). Subsequently, two bit streams are independently recovered as two pictures (picture I and picture II), but both of them are meaningless, thus protecting the information security.

**Figure 4:**
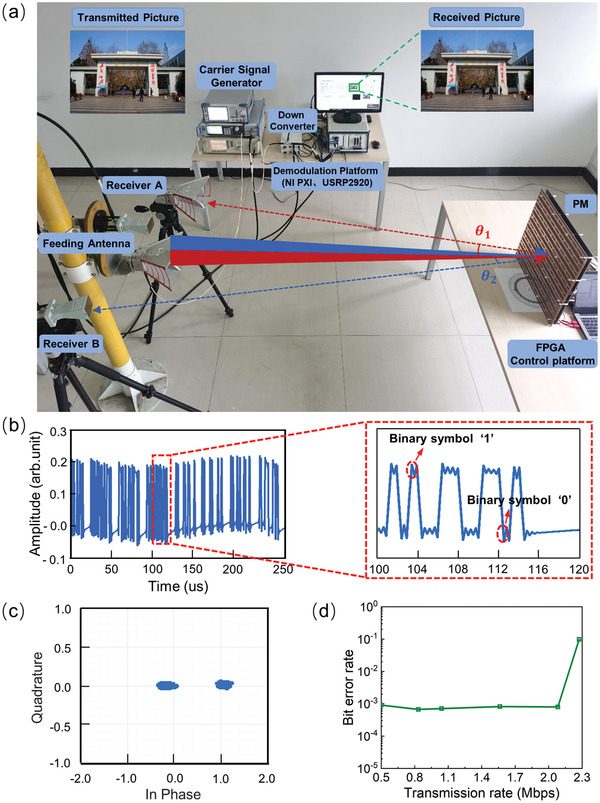
a) The fabricated dual‐channel wireless communication system. b) Under the transmission rate of 2 Mbps, the selected demodulated signal with about 250 µs length at receiver A, in which the magnitude of the signal amplitude represents the binary symbol “0” or “1”. Further, the signal parts within the range of 100–120 µs marked in red are enlarged at the right side. c) The measured constellation diagram of the wireless communication system with a transmission rate of 1.563 Mbps. d) The variation trend of a single channel's bit error rate with the change of transmission rate.

A realistic wireless communication system is built to perform proof‐of‐concept validation in an indoor scenario, as depicted in **Figure** **4**a. The constructed system comprises a transmitter and a receiving terminal. The transmitter is mainly composed of the FPGA control platform, the fabricated PM and two linearly polarized horn antennas connected to carrier wave generators. The receiving terminal contains the other two linearly polarized receiving antennas (2.5 meters away from the PM), the down‐converter and the demodulation platform (USRP 2920 and NI control platform PXIe‐1092, National Instruments Corp.). Receiver A and receiver B are located at directions with *θ*_1_ = −12° and *θ*_2_ = 5° with respect to the surface normal of the PM, respectively.

In the experiment, a color picture is divided and transformed into two control signals (grouping number *n* = 10 000) to drive the PM by the process described in Figure 3a. Two carrier waves (baseband signals) at frequencies of *f*_1_ = 2.15 GHz and *f*_2_ = 5.34 GHz illuminate on the PM via two linearly polarized horn antennas and are then modulated by the PM and scattered towards the receiving directions. The other two horn antennas, serving as receiver A (*f*_1_) and receiver B (*f*_2_), are used to receive the modulated baseband signals independently and then transmit to the USRPs. In detail, the received signal at receiver B is first converted to a lower frequency *f*_3_ = 1 GHz through a down‐converter and then transmitted to one USRP, while the signal at receiver A is directly transmitted to the other USRP. After that, the modulated baseband signals of *f*_1_ and *f*_3_ are demodulated into binary symbols via threshold decision. The demodulated signal of *f*_1_ with a length about 250 µs is depicted in Figure 4b. From the enlarged part at right side, it is clear that two different magnitudes of the signal amplitude represent the demodulated binary symbol “0” and “1”, respectively. For example, the signal of the enlarged part represents the demodulated bit stream of “001 100 110 000 111 10000…”. At last, the demodulated signals of *f*_1_ and *f*_3_ are synthetized in order via the NI control platform to retrieve the transmitted picture in real time, as shown in the inset of Figure 4a.

The picture is fully recovered at the receiving terminals by combining their received information, and the BER is as low as 8.1×10^−4^ compared with the original picture. To check the modulation scheme of the transmitter, the measured constellation diagram that represents the 1‐bit binary symbols is provided in Figure 4c, in which the distance of each point to the origin indicates the reflection amplitude of the binary symbol. Here, two constellation points are observed in the diagram, representing binary symbols “0” and “1”, thus verifying the BASK modulation. As shown in Figure 4d, the measured BER of a single channel remains at approximately 8.2×10^−4^ until the transmission rate is more than 2.083 Mbps, which means that each single channel works very well with a maximum transmission rate of about 2.083 Mbps. More details about the communication performance of a single channel are provided in Figure S5 in the Supporting Information. Therefore, the total transmission rate can reach up to 4.167 Mbps while keeping a low BER. Other key parameters of the designed system are listed in **Table** **1**. The communication performance of the system will gradually degrade when the transmission distance is larger than 5 m for the current scheme. Further increase of the distortionless transmission range can be achieved by enhancing the emitting power or applying channel coding to improve the accuracy of the demodulated information. Besides, the received signal of each receiver cannot be individually demodulated into correct information. In addition, when receiver A or B moves to undesired directions (for example, receiver A moves to *θ* = −5°), the synthetized signals from two physical channels cannot be correctly demodulated even if the transmitting power is substantially increased.

**Table 1:** Key parameters of the established wireless communication system

Key Parameters of the Wireless Communication System	
Parameter	Value
Working frequency	2.15 GHz / 5.34 GHz
Frame size	10 000 samples
Image size	220×154 pixel
Bit error rate	8.1×10^−4^
Transmit power	5 dBm
Receiver gain	20 dB
Transmission distance	4.5 meter
Transmission rate	4.167 Mbps

To further demonstrate the reprogrammable and high‐security features of the proposed wireless communication system, another experiment is carried out with receivers A and B located in the same direction, as illustrated in Figure S6 in the Supporting Information. The details of how the system change from one case to the other is described in Figure S7 in the Supporting Information. Here, receiver A and receiver B are both located at *θ* = −12° with a slight altitude difference to avoid the blockage of each other, while the other configurations of the system are kept unchanged. The transmitted picture is also successfully retrieved via two receivers, revealing that the two receivers have their own independent receiving channels via different frequencies and will not trigger the interference issue between two receivers located at the same position. Hence, whether in remote directions out of the beam or in the same directions as the receivers, the complete transmitted information cannot be intercepted and deciphered by a single‐band eavesdropper. Moreover, owing to the advantage of independently designed working frequencies, the two channels could be in different 5G communication frequencies (for example, one channel in n1 band and the other in n79 band). Thus, the system also has the advantage of resisting external electromagnetic interference, for example, that from signal blockers or jammers, because when one frequency channel is blocked, the other may still work. The phase manipulation based on 1‐bit metasurface (0 and *π*) has its own limitations in beam generation due to the phase quantization. For example, when the beam angles increase, the beam patterns will deteriorate and the side lobes will become larger. Such problems may be solved by multilevel phase‐programmable metasurface or by adopting proper algorithm to optimize the beam performances.

## 5 Conclusion

We have proposed a metasurface‐assisted wireless communication scheme in which the transmitter does not include complicated mixing and RF modules but is based on a programmable metasurface that directly modulates the carrier waves to encode the digital information at two discrete frequencies. The proposed scheme is implemented to construct a dual‐channel wireless communication system for information security. Experiments demonstrate that a color picture can be encrypted and transmitted through two physical communication channels to two legitimate receivers, independently and simultaneously, and then retrieved in the demodulation platform. The overall system performance could be further enhanced by optimizing the metasurface with a multilevel responses and using the PIN diodes with higher switching speed. The proposed communication scheme not only has low cost and low complexity but also offers a new approach to improve the information security for emerging direct wireless communication systems, which may have a positive impact on next‐generation communication systems and radar systems. Moreover, the current designs may also be extended to other frequencies, such as millimeter waves and broadcast bands, to obtain more extensive applications.

## Conflict of Interest

The authors declare no conflict of interest.

## Supporting information

Supporting Information

## Data Availability

The data that support the findings of this study are available from the corresponding author upon reasonable request.
