# Supplementary material for: Metasurface‐Assisted Wireless Communication with Physical Level Information Encryption
Source: Adv Sci (Weinh). 2022 Oct 17;9(34):2204558. doi: 10.1002/advs.202204558 (PMC9731697; doi:10.1002/advs.202204558)
Supplement: Supplementary file 1 — Supporting Information [file ADVS-9-2204558-s001.pdf]

ADVANCED SCIENCE
Open Access

Supporting Information

for *Adv. Sci.*, DOI 10.1002/advs.202204558

Metasurface-Assisted Wireless Communication with Physical Level Information Encryption

Yilin Zheng, Ke Chen, Zhiyuan Xu, Na Zhang, Jian Wang, Junming Zhao and Yijun Feng**

Supporting Information

Metasurface-assisted wireless communication with physical level information encryption

Yilin Zheng, Ke Chen, Zhiyuan Xu, Na Zhang, Jian Wang, Junming Zhao, and Yijun Feng**

1. Biasing network design of the meta-atom

On the top layer, the bias neatwork is etched on the surface, as shown in **Figure S1**(a). The positive poles of each column are connected to independent wiring ports located at bottom end of the top layer through metal vias and etched lines on the back of top layer. The negative poles of each column are connected to an additional metal patch, which can be regarded as the ground of the top layer. Unlike the top layer, the structure of the bottom-layer consists of three metallic layers and two dielectric layers, in which nine metallic via holes are drilled through the substrates to connect the I-shaped patterns with nine pieces of connected patches on the bottom metallic layer (Figure S1(b)) that are used as the positive electrodes to apply the direct current (DC) bias voltage. The longer metal arms on the bottom layer are connected to the middle metallic ground plane, which are used as the negative electrodes. In this way, the top layer and bottom layer of the meta-atom can be biased by the DC voltage independently and simultaneously.

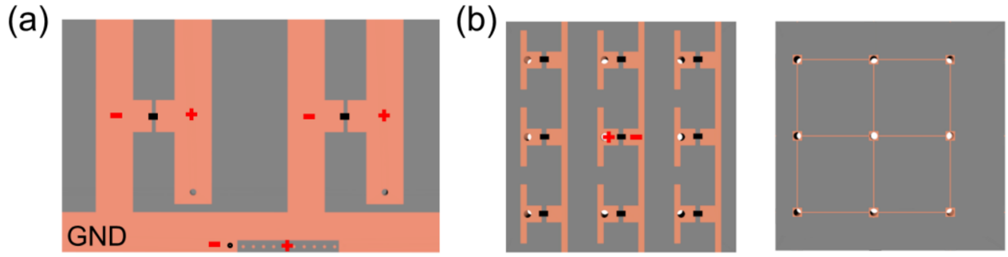

Figure S1. Schematic of the bias network design for the (a) top layer and (b) bottom layer of the dual-band programmable meta-atom.

2. Additional properties of the meta-atom in simulation

In order to further demonstrate that the two channels can be manipulated independently, the additional reflection amplitude and phase responses of the meta-atom are shown here. As depicted in **Figure S2**(a), a phase difference of 180° and reflection amplitude over 0.95 can be obtained at 2.4 GHz when the PIN diodes on the top layer are switched between 'ON' to

'OFF' states while the PIN diodes on the bottom layer are kept in 'ON' state. Similar results can be observed at 5 GHz when the PIN diodes on the bottom layer are switched between 'ON' to 'OFF' states while the PIN diodes on the top layer are kept in 'ON' state, as shown in Figure S2(b). Combined with the reflection responses shown in Figure 2(b) and 2(c), it's clear the amplitude and phase responses of each layer of the meta-atom are kept the same, regardless that the PIN diodes on the other layer are at 'ON' or 'OFF' state. Therefore, the two frequency-channels are completely independent.

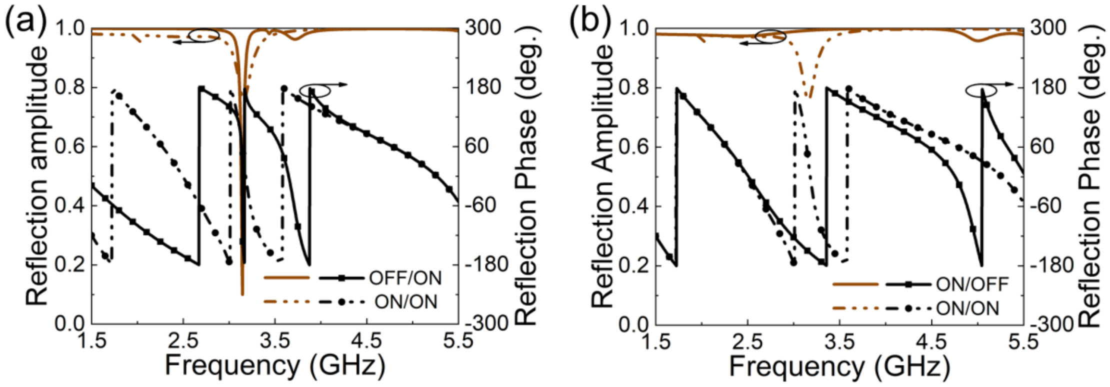

Figure S2. (a) The simulated reflection responses to the 'ON' and 'OFF' states of the PIN diodes on the top layer when the PIN diodes on the bottom layer are in the 'ON' state. (b) The simulated reflection responses to the 'ON' and 'OFF' states of the PIN diodes on the bottom layer when the others on the top layer are in the 'ON' state.

3. More cases for receivers located in different directions

In the proposed wireless communication system, the receivers could be at any directions in the reflection space of the metasurface by applying different sets of coding matrices to drive the designed programmable metasurface (PM). Figure S3 displays examples for the simulated far-field patterns of different optimized coding matrices for receiver A (at f_1 = 2.4 GHz) and receiver B (at f_2 = 5 GHz), respectively. In **Figure S3**(a), the main beam of $M_{1\text{-}3}$ has a strong peak in the direction of θ_1 = 19° and transmits binary symbol '1' to receiver A, while $M_{1\text{-}4}$ produces a weak beam in the same direction and transmits binary symbol '0' to receiver A. In Figure S3(b), the main beam of $M_{1\text{-}5}$ transmits binary symbol '1' to receiver A in the direction of θ_1 = 30°, while the beam of $M_{1\text{-}6}$ transmits binary symbol '0'. As for the other physical channel of 5 GHz, the radiation beam of optimized coding matric $M_{2\text{-}3}$ has a strong peak in the direction of θ_2 = 12° and transmits binary symbol '1' to receiver B, while the beam of $M_{2\text{-}4}$ transmits binary symbol '0' in the same direction, as shown in Figure S3(c). At the same time, the simulated far-field patterns for the other transmission direction of θ_2 = 20° are also depicted in Figure S3(d), in which the main beam of $M_{2\text{-}5}$ transmits binary symbol '1' to

receiver B while the beam of $M_{2\text{-}4}$ transmits binary symbol '0'. For the proposed wireless transmission system, different values of θ_1 and θ_2 can be combined into different cases of information decryption, for example, (θ_1, θ_2) = (19°, 12°) and (30°, 20°). Actually, combined with the far-field patterns depicted in Figure 2, there are totally nine cases of the proposed wireless communication system since the transmission directions at each channel can be used in any combination of two. Nevertheless, the values of θ_1 and θ_2 are not limited to the examples here and have many other options in the reflection space; thus there are much more cases for receivers located in different directions.

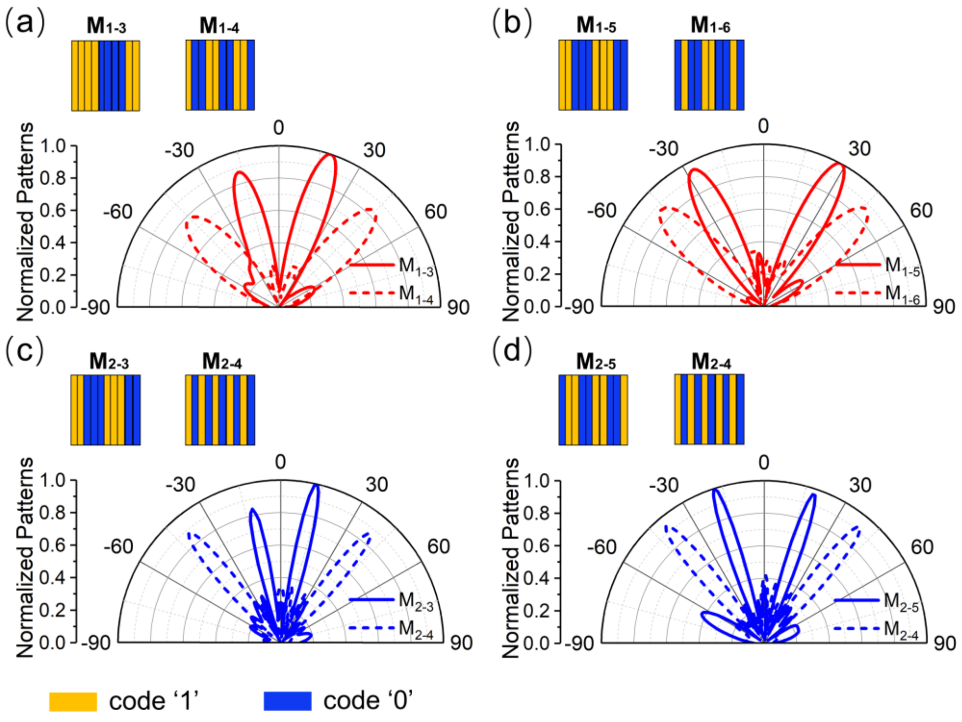

Figure S3. The simulated far-field patterns of the PM for receivers at different directions. Simulated patterns of optimized coding matrices (a) $M_{1\text{-}3}$ and $M_{1\text{-}4}$ for receiver A (at f_1 2.4 GHz) in the direction of θ_1 = 19°; (b) $M_{1\text{-}5}$ and $M_{1\text{-}6}$ for receiver A (at f_1 2.4 GHz) in the direction of θ_1 = 30°; (c) $M_{2\text{-}3}$ and $M_{2\text{-}4}$ for receiver B (at f_2 5 GHz) in the direction of θ_2 = 12°; (d) $M_{2\text{-}5}$ and $M_{2\text{-}4}$ for receiver B (at f_2 5 GHz) in the direction of θ_2 = 20°.

4. Properties of the meta-atom in experiment

Experiments are also carried out to measure the electromagnetic (EM) responses of the fabricated sample. All the PIN diodes embedded on the same layer of the metasurface are biased by the same voltage and controlled by FPGA to shift between 'ON' (3.3 V) and 'OFF' (0 V) states. PIN diodes on different layers are independently controlled. When the metasurface is illuminated by *y*-polarized EM wave, the reflection amplitude and phase responses of the meta-atom are illustrated in **Figure S4**(a) and S4(b). It can be observed that a

phase difference of 180° and reflection amplitude over 0.7 at 2.15 GHz can be obtained when the PIN diodes on the top layer are switched between 'ON' and 'OFF' states while those on the bottom layer are kept as 'OFF' state. Similarly, a phase difference of 170° and reflection amplitude over 0.6 can be observed at 5.34 GHz when the PIN diodes on the bottom layer are switched from 'ON' to 'OFF', as shown in Figure S4(b). Though the measured reflection amplitudes decrease a little compared with the simulation results, the measured phase difference are enough to realize different coding matrices. The amplitude deterioration might come from the loss of the PIN diodes and the assembly tolerance.

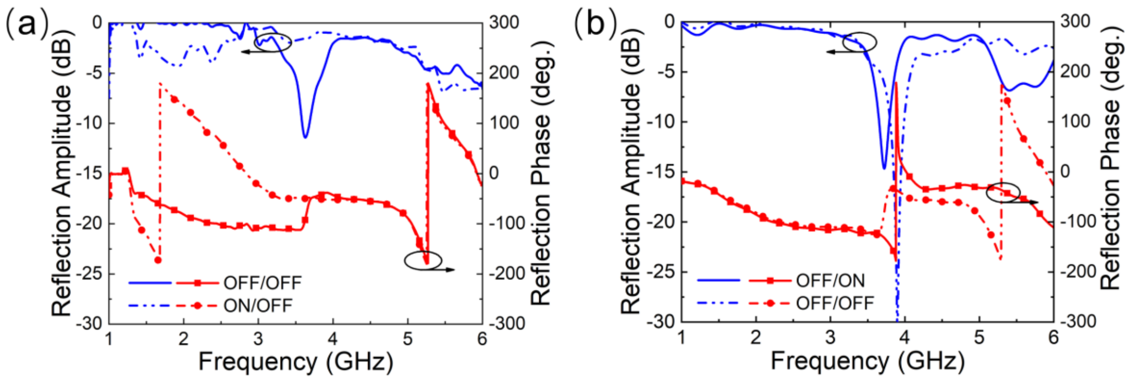

Figure S4. (a) The measured reflection responses to the 'ON' and 'OFF' states of the PIN diodes on the top layer when the PIN diodes on the bottom layer are in the 'OFF' state. (b) The measured reflection responses to the 'ON' and 'OFF' states of the PIN diodes on the bottom layer when the others are in the 'OFF' state.

5. Transmission performance of each physical channel

In order to evaluate the transmission performance of each physical channel, two pictures are transmitted through two channels independently. Different from the information-encryption experiment, two pictures are independently translated into two control signals to drive the PM, and the received signals at receiver A (θ = -12°) and receiver B (θ = 5°) are demodulated individually to recover two transmitted pictures. It turns out that two pictures are recovered successfully at the same time with a low bit error rate of 6.7×10^{-4} and a maximum transmission rate of 2.083 Mbps. Moreover, the two receivers are moved around the pre-set direction to investigate the receiving range of each channel. As depicted in **Figure S5**(a), receiver A can successfully retrieve the transmitted picture from θ = -14° to θ = -9° (bit error rate is lower than 1.0×10^{-2}), while received signals cannot be demodulated as the correct transmission information at other remote directions, demonstrating that the receiving range of channel A is about 5° around center angle θ = -12°. Similarly, the measured receiving range of channel B is also about 5° around center angle θ = 5°, as shown in Figure S5(b). As a result,

the receiving ranges of two channels accord well with the previously measured far-field patterns of the PM (depicted in Figure 2(h) and (i)), which further illustrate the good directivity and privacy of the transmission channels.

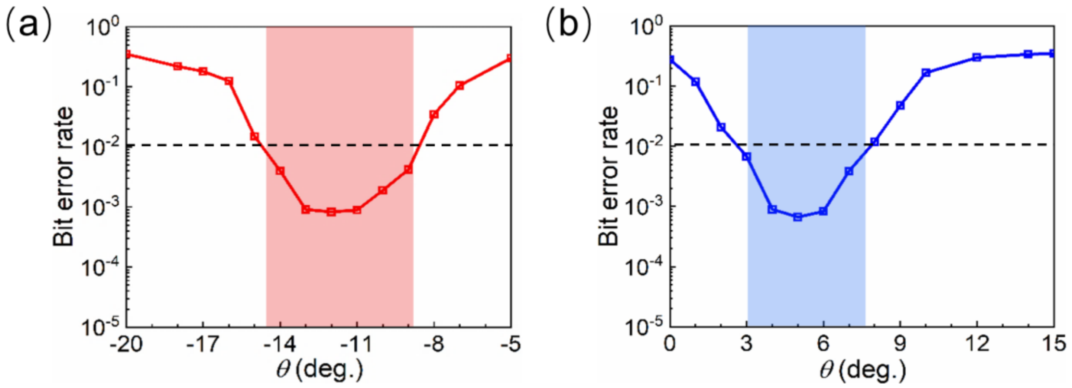

Figure S5. Transmission performance of each physical channel in experiment. (a) The receiving range schematic of receiver A (f_1 = 2.15 GHz). (b) The receiving range schematic of receiver B (f_2 = 5.34 GHz).

6. Experiment scenario for receivers in the same direction

In addition, to further demonstrate the reprogrammable and high-security features of the proposed wireless communication system, another experiment is carried out with receiver A and B located in the same direction. As shown in **Figure S6**, receiver A and receiver B are both located at θ = -12° with a slight altitude difference, while other configurations of the system are kept the same with the experimental scenario described in Figure 4. Two carrier waves (baseband signals) at frequencies of f_1 = 2.15 GHz and f_2 = 5.34 GHz are illuminated on the PM in the meantime and then modulated by the control signals from the FPGA. At the receiving terminal, the modulated baseband signals are received by receiver A (f_1) and receiver B (f_2) independently and then transmit to the USRPs. Among them, the received signal at receiver B is first converted to a lower frequency f_3 = 1 GHz through a downconverter and then transmitted to one USRP, while the signal at receiver A is directly transmitted to the other USRP. At last, the modulated baseband signals of f_1 and f_3 are demodulated and synthetized in order to retrieve the transmitted picture (as shown in the inset of Figure S6) in real time with a BER of 8.7×10^{-4}. The experiment result indicates that the two channels located in the same direction won't interfere with each other and still with good confidentiality. Furthermore, it also proves that the complete transmitted information cannot be deciphered by a single-band eavesdropper even when it's at the same location with two encryption channels, revealing the high security of the wireless communication system.

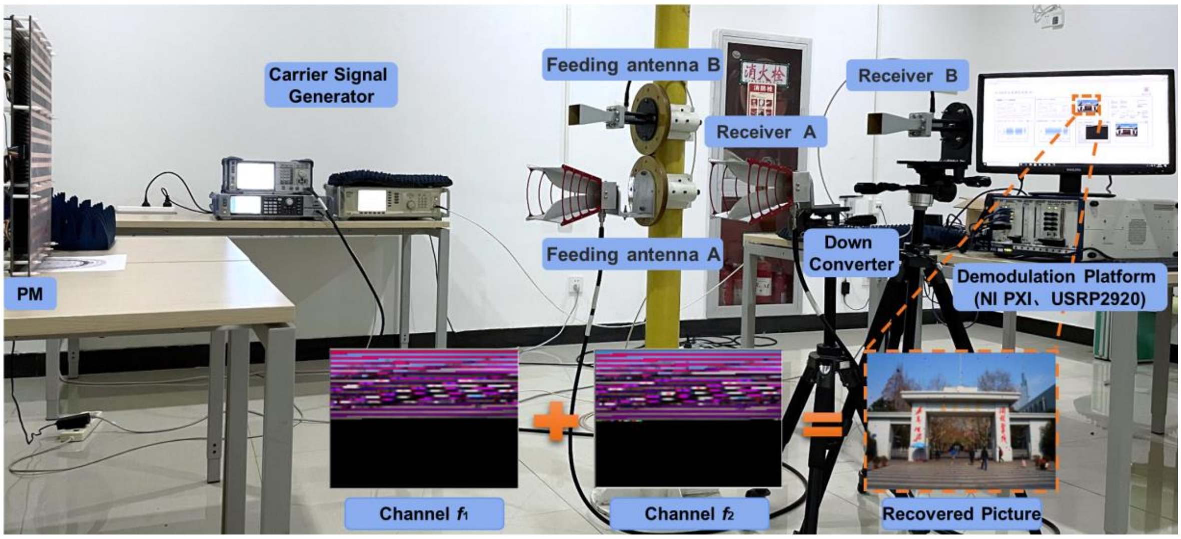

Figure S6. The constructed dual-channel wireless communication system with two receivers in the same direction.

7. Realization of dynamic wireless communication system

A flow chart is employed in **Figure S7** to show the dynamic change of the wireless communication system when the azimuth angle θ is changed. Firstly, the variable azimuth angle θ is input to the pre-designed program to calculate the required coding matrixes applied onto the metasurface. Then, the corresponding control signals are generated by the FPGA based hardware controller to drive the PIN diodes on meta-atoms, and thus form the corresponding phase profile on the metasurface aperture. Finally, the reflection beams are generated by the programmable metasurface to construct transmission channels at different directions. It's worth mentioning that the hardware controller can simultaneously output hundreds signals of bias voltage. Therefore, all the PIN diodes on the metasurface can be controlled at the same time. When the receiver direction is changed, the metasurface can be adjusted to point at the receiver through the process mentioned above.

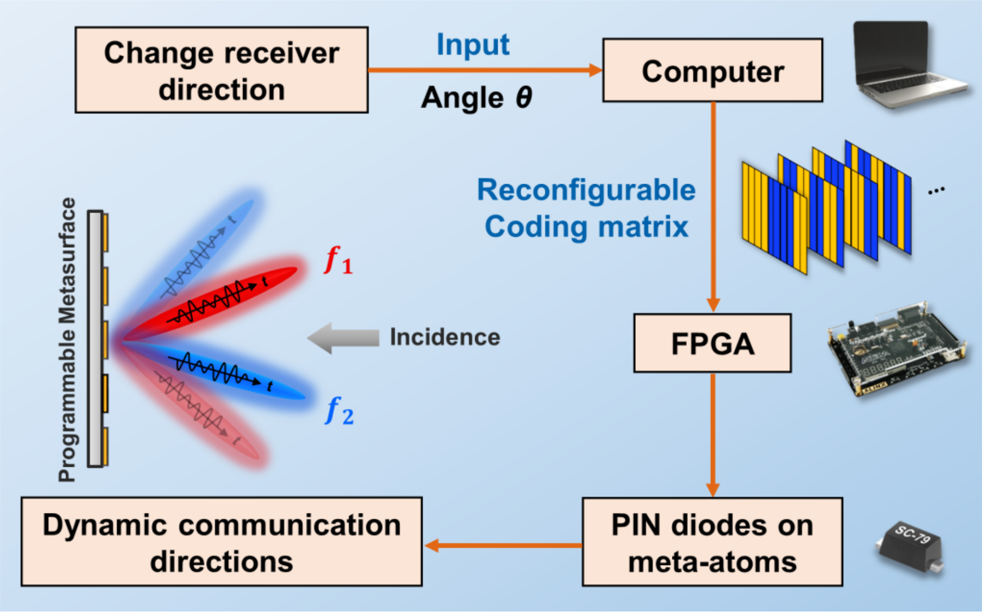

Figure S7. Flow chart of the realization of dynamic wireless communication system
